# Association of accelerated dynamics of telomere sequence loss in peripheral blood leukocytes with incident knee osteoarthritis in Osteoarthritis Initiative cohort

**DOI:** 10.1038/s41598-021-95326-7

**Published:** 2021-08-05

**Authors:** Rebeca Guillén, Fátima Otero, Alejandro Mosquera, María Vázquez-Mosquera, Ignacio Rego-Pérez, Francisco J. Blanco, José Luis Fernández

**Affiliations:** 1 Genetics Unit, INIBIC-Complexo Hospitalario Universitario A Coruña (CHUAC), A Coruña, Spain; 2 Laboratory of Genetics and Radiobiology, Centro Oncológico de Galicia, A Coruña, Spain; 3 Rheumatology Division, INIBIC-Complexo Hospitalario Universitario A Coruña (CHUAC), A Coruña, Spain; 4 Department of Physiotherapy, Medicine and Biomedical Sciences, Strategic Group CICA-INIBIC, Rheumatology and Health Group, Universidad de A Coruña, A Coruña, Spain; 5 Rheumatology Division, Complexo Hospitalario Universitario A Coruña (CHUAC), As Xubias, 84, 15006 A Coruña, Spain

**Keywords:** Ageing, Physiology, Rheumatology

## Abstract

Osteoarthritis (OA) is a chronic degenerative joint disease, being the main cause of laboral inability. Decreased telomere size in peripheral blood leukocytes (PBL) has been correlated with age-related pathologies, like knee OA. In a dynamic approach, telomere-qPCR was performed to evaluate the relative percentage of PBL telomere loss after a 6-year follow-up, in 281 subjects from the prospective osteoarthritis initiative (OAI) cohort. A radiological Kellgren-Lawrence (KL) grade ≥ 2 was indicative of knee OA. Individuals with knee OA at recruitment (n = 144) showed a higher PBL telomere loss after 6 years than those without knee OA at baseline (n = 137; *p* = 0.018). Moreover, individuals that developed knee OA during the follow-up (n = 39) exhibited a higher telomere loss compared to those that remained without OA (n = 98; *p* < 0.001). Logistic regression analysis showed that PBLs telomere loss was not significantly associated with knee OA at recruitment, but behaves as an independent risk factor associated with incidence after follow-up (OR: 1.043; *p* = 0.041), together with maximum KL grade (OR: 3.627; *p* = 0.011), body mass index-BMI (OR: 1.252; *p* < 0.001) and WOMAC-index (OR: 1.247; *p* = 0.021), at recruitment. The telomere decay in PBLs is faster in individuals with incident knee OA, possibly reflecting a systemic-global accelerated aging that enhances the cartilage degeneration.

## Introduction

Osteoarthritis (OA) is the most common pathology of cartilage and joints, being a major cause of labor inability. Chondrocyte dysfunction possibly related to premature senescence leads to extracellular matrix degradation and local secretion of proinflammatory cytokines^1,2^.

Telomeres are chromatin structures that cup the end of eukaryotic chromosomes. Human telomere DNA is constituted by a tandem array of the sequence 5’-TTAGGG-3’ and the complementary strand 5’-CCCTAA-3’. Every time a cell replicates the DNA, 50–150 pb of terminal telomeric DNA sequence are lost, so the telomere is progressively shorter with aging, in somatic cells. If a critical size is achieved, replicative senescence is triggered, so the cell stops dividing and may die by apoptosis^3,4^.

The telomere size from human peripheral blood leukocytes (PBL) decreases with aging. The length of telomeres from leukocytes correlates with that from other somatic tissues from the same subject, independently of their proliferative activity^5,6^. Telomere length evaluation has been found to be indicative of biological age and a potential predictor of lifespan in human populations^7^. Furthermore, decreased PBL telomere size consistently correlated with increased risk of several common age related pathologies, like cardiovascular diseases, some cancer types, type II diabetes and dementia^4,8,9^.

It has recently been demonstrated in individuals from the Osteoarthritis Initiative (OAI) consortium from USA that mean PBL telomere size at recruitment was an independent risk factor for concurrent knee OA, as well as radiological severity^10^. Furthermore, this baseline PBL telomere length was associated with incident hand OA^11^.

All the evaluations of PBL telomere size in OA studies were established at a single time-point. Nevertheless, telomere sequences decrease with time. The rates of decay in telomere dynamics could be more informative and relevant than single time-point determinations. The OAI consortium has been prospectively collecting accurate and methodical data during several years of follow-up, including detailed clinical, imaging and analytical parameters^12^. This OAI cohort is of choice to try to determine how PBL telomeres evolve in the same individual, in relation to OA.

## Results

### Cohorts characteristics

281 individuals (152 women) of Caucasian ancestry were eligible for the study based on availability of both baseline and 72-month radiographs and baseline PBL telomere length data. Mean age was 58.48 ± 7.27 years. Among the 281 individuals at baseline, 144 met the definition for prevalent knee OA and 137 for non-prevalent (without knee OA). After 6 years of follow-up, those prevalent at baseline continued with knee OA, as is obvious. On the other hand, focusing on those non-prevalent at baseline, after 6 years of follow-up, 39 developed knee OA (incident) whereas 98 continued without knee OA (non-incident) (Table 1).

**Table 1:** Characteristics of the cohorts.

		Non-incident (n = 98)	Incident (n = 39)	Prevalent (n = 144)	Total (n = 281)
Age (mean ± SD)		56.85 ± 7.52	59.03 ± 6.97	59.44 ± 7.04	58.48 ± 7.27
Gender (n (%))	Female	60 (61.22)	26 (66.67)	66 (45.83)	152 (54.09)
	Male	38 (38.78)	13 (33.33)	78 (54.17)	129 (45.91)
BMI (mean ± SD)		25.00 ± 3.54	29.66 ± 4.28	29.05 ± 4.35	27.72 ± 4.53
Hypertension (n (%))	Yes	19 (19.39)	9 (23.08)	45 (31.25)	73 (25.98)
	No	79 (80.61)	30 (76.92)	99 (68.75)	208 (74.02)
Baseline max-KL (n (%))	0	69 (70.41)	11 (28.21)	0 (0.00)	80 (28.48)
	1	29 (29.59)	28 (71.79)	0 (0.00)	57 (20.28)
	2	0 (0.00)	0 (0.00)	78 (54.17)	78 (27.76)
	3	0 (0.00)	0 (0.00)	48 (33.33)	48 (17.07)
	4	0 (0.00)	0 (0.00)	18 (12.50)	18 (6.41)
% PBL telomere loss (mean ± SD)		27.70 ± 13.25	38.51 ± 9.33	34.37 ± 11.61	32.62 ± 12.50
WOMAC (mean ± SD)		0.95 ± 2.29	3.52 ± 3.41	9.94 ± 5.99	3.86 ± 5.19

### Prevalent radiographic knee OA

Individuals with prevalent knee OA at recruitment (max-KL ≥ 2; n = 144) showed a significant higher relative percentage of PBL telomere loss after 6 years than those without radiographic knee OA at baseline (max-KL < 2; n = 137; median: 35.05 vs 30.44, *p* = 0.018) (Table 2A). Moreover, PBL telomere shortening tended to be faster in males compared to females (*p* = 0.094). No significant differences were observed between individuals with or without hypertension (*p* = 0.318) (Table 2A). A positive correlation was found between PBL telomere loss and BMI (*p* = 0.010) (Table 2B). The correlation between age and PBL telomere decay was near the limit of statistical significance (*p* = 0.091) (Table 2B).

**Table 2:** Evaluation of the relative percentage of telomere loss after 6 years of follow-up, in peripheral blood leukocytes from individuals from the OAI cohort, in relation to parameters obtained at recruitment. **(A)** Comparisons performed by Mann–Whitney U-test. **(B)** Correlation analysis.

			n	Mean	95% CI	Median	*P*
**(A)**	Gender	Male	129	33.98	31.68–36.29	35.76	0.094
		Female	152	31.47	29.58–33.35	31.82	
	Hypertension	Non-hypertension	208	32.21	30.54–33.89	32.10	0.318
		Hypertension	73	33.78	30.72–36.85	35.24	
	Baseline max-KL grade	KL < 2	137	30.78	28.55–33.00	30.44	0.018
		KL ≥ 2	144	34.37	32.46–36.29	35.05	
**(B)**	Age (years)	Spearman’s Rank Correlation Coefficient					0.091 (r = 0.101)
	BMI (Kg/m^2^)						0.010 (r = 0.153)

Multivariable analysis by binary logistic regression analysis did not reveal any significant association of age, hypertension and dynamics of PBL telomere loss with prevalent knee OA (Table 3A). Yet, BMI (OR: 1.071; *p* = 0.042), gender (male; OR: 0.577; *p* = 0.048) and Western Ontario and McMaster Universities Osteoarthritis Index (WOMAC) (OR: 1.273; *p* < 0.001) manifested as risk factors significantly associated with prevalent knee OA (Table 3A). The three previously identified significant variables, BMI, gender and WOMAC-index, were introduced in the logistic regression model, maintaining their significance (Table 3B).

**Table 3:** Prevalent and incident radiographic knee OA: (A) Multivariable analysis using logistic regression analysis to evaluate the association of variables with prevalent and incident knee osteoarthritis. (B) Logistic regression model with selected variables. Significance was defined as *p* < 0.05.

		Prevalent			Incident		
		OR	95% CI	*P*	OR	95% CI	*P*
**(A)**	Age (years)	1.030	0.991–1.070	0.134	1.041	0.971–1.115	0.257
	Baseline max-KL grade	–	–	–	3.988	1.420–11.196	**0.009**
	BMI (Kg/m^2^)	1.071	1.002–1.144	**0.042**	1.266	1.115–1.437	** < 0.001**
	Gender (Male)	0.577	0.334–0.995	**0.048**	2.534	0.872–7.364	0.088
	Hypertension	1.397	0.746–2.616	0.296	0.518	0.144–1.858	0.313
	% PBL telomere loss	1.011	0.989–1.034	0.313	1.047	1.002–1.094	**0.039**
	WOMAC	1.273	1.162–1.395	** < 0.001**	1.286	1.065–1.552	**0.009**
**(B)**	Baseline max-KL grade	–	–	–	3.627	1.342–9.800	0.011
	BMI (Kg/m^2^)	1.082	1.013–1.155	0.019	1.252	1.106–1.417	< 0.001
	Gender (Male)	0.558	0.325–0.956	0.034	–	–	–
	% PBL telomere loss	–	–	–	1.043	1.002–1.087	0.041
	WOMAC	1.280	1.164–1.396	< 0.001	1.247	1.034–1.505	0.021

### Incident radiographic knee OA

**Figure 1:**
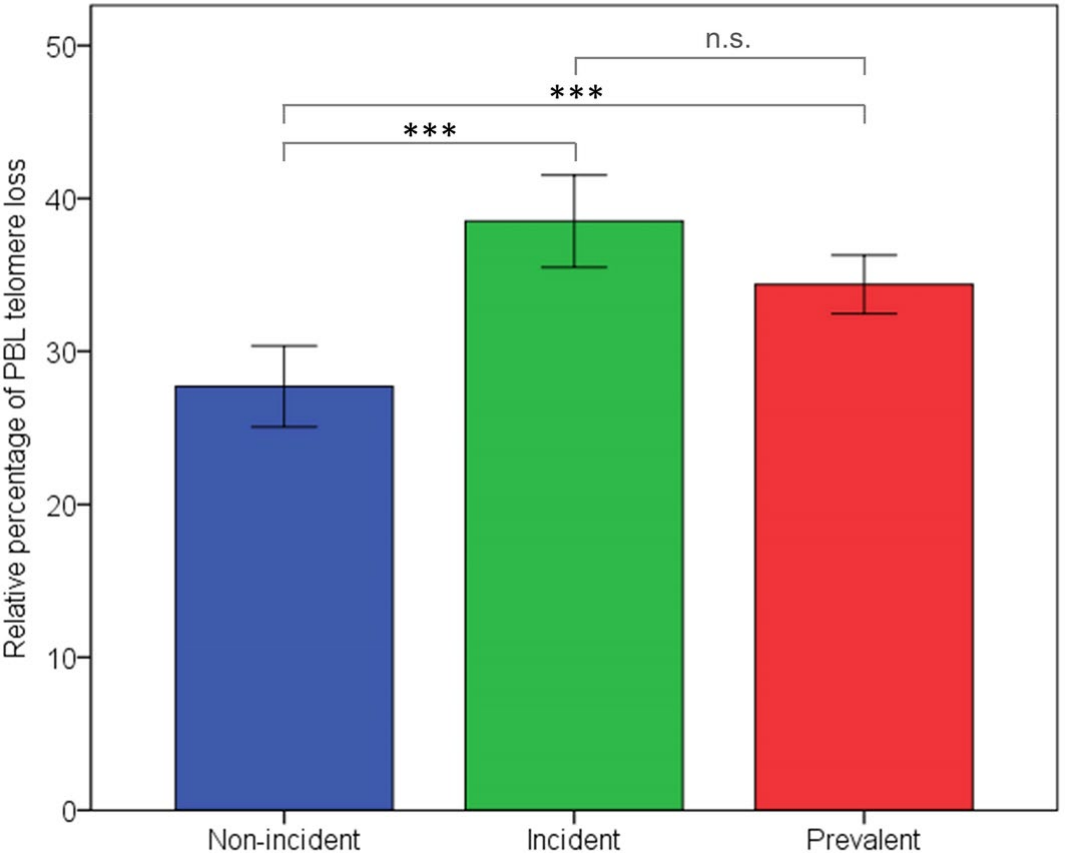
Rate of PBL telomere loss in prevalent, incident and non-incident knee OA. Kruskal–Wallis test evidenced significant differences among the three groups (p < 0.001). Incident knee OA exhibited a higher relative fraction of PBL telomere loss in comparison to non-incident OA (mean: 38.51 vs 27.70, Mann–Whitney U-test, *p* < 0.001). Magnitude of dynamic loss was not significantly different between patients with incident and prevalent knee OA (mean: 38.51 vs 34.37, Mann–Whitney U-test,* p* = 0.142). (Significance *p* value: *** < 0.001; n.s.: not significant).

Regarding incident knee OA, those individuals that developed radiographic knee OA during the 6 years of follow-up period (n = 39), exhibited a higher relative percentage of PBL telomere loss in comparison to those that remained without knee OA (n = 98; median: 41.36 vs 26.25, *p* < 0.001). However, their magnitude of dynamic loss was not significantly different from that of patients with prevalent knee OA (median: 41.36 vs 35.05 *p* = 0.142) (Fig. 1).

Binary logistic regression analysis evidenced that the dynamics of PBL telomere loss was a significant associated risk factor of incidence of knee OA (OR: 1.047; *p* = 0.039), as well as BMI (OR: 1.266; *p* < 0.001), WOMAC (OR: 1.286; *p* = 0.009) and max-KL grade (OR: 3.988; *p* = 0.009), at baseline (Table 3A). Age, gender and hypertension were not found to be significantly associated. Under the logistic regression model including the four variables previously recognized as significant, PBL telomere decay remained significantly associated with incident knee OA (OR: 1.043; *p* = 0.041) (Table 3B).

## Discussion

Assessment of telomeres in PBL provides knowledge about how systemic biological aging is progressing and subsequently may indicate how the articular senescence level is evolving. The present study intended to perform the first evaluation of the dynamics of telomere decay in each individual of the selected cohort. This is a more stringent assessment than the single-time point evaluation and confidently confirms a higher velocity of PBL telomere loss in individuals with prevalent and with incident knee OA.

Although PBL telomere size may be associated with many parameters related to aging and health status, multivariable analysis using logistic regression analysis had evidenced PBL telomere size at recruitment as a risk factor significantly associated to the prevalence of knee OA at baseline^10^. Regarding hand OA, the association of PBL telomere amount at recruitment with prevalent hand OA lost significance in the model adjusted for age, sex and BMI, whereas the association was evidenced for incident hand OA^11^. The results of our dynamic telomere approach were close to those from the single-time point evaluation at baseline regarding hand OA. In fact, the relative percentage of PBL telomere decay did not reach statistical significance as an independent risk factor for prevalent knee OA, whereas it was a significantly associated risk marker of incident knee OA.

It seems paradoxical that the single-time determination of PBL telomeres at recruitment was significantly associated with concurrent knee OA, whereas the dynamic telomere decay did not. Nevertheless, despite the relative percentage of PBL telomere loss in our group of individuals with prevalent knee OA, it was lower than that compared to incident OA, it was higher in comparison with non-incident subjects, although not reaching statistical significance. A relative highly accelerated PBL telomere decay may indicate a rapid progression of biological aging at the systemic level, which locally, at the joint cartilage, may be related to accelerated chondrocyte senescence and subsequent development of knee OA, i.e. incidence. Once the illness is established, the telomeres would be eroded at a lower rate. In fact, the standard PBL telomere decay is not homogeneous throughout life, being more intense during initial years and slowing slightly with aging^13^. Since biological aging may be accelerated in OA, the progressive slowing kinetics could be shifted to earlier chronological ages.

Overall, knee OA is mainly developed in the group of subjects with a higher PBL telomere loss over time, i.e. with an accelerated biological aging phenotype^14^. This unfavourable physiological background would enhance chondrocyte senescence, decreasing the cartilage resistance to other mechanic, metabolic, inflammatory or oxidative stressors^15^. Some of these stressors may be in connection with risk factors evidenced in the logistic regression analysis, like BMI, which may be linked to obesity. The confluence of all these factors would contribute to the appearance and progression of the articular destruction. This was a retrospective cohort study, limited to a subset of Caucasian subjects and results support a further validation using prospective cohorts. In addition, the sample size of individuals with incident knee OA is not very high, but necessarily related to the natural evolution of the pathology itself after the selection of healthy individuals.

## Patients and methods

### Subjects

To study the association between radiographic knee OA and telomere length we conducted a cross-sectional and longitudinal analysis of data from a subset of participants in the Osteoarthritis Initiative (OAI). The OAI is a multicenter cohort study of 4,796 adults with or at risk for symptomatic knee OA. Four clinical sites (Memorial Hospital of Rhode Island, The Ohio State University, University of Maryland and Johns Hopkins University, and the University of Pittsburgh) recruited participants between February 2004 and May 2006. Three subcohorts are identified; Progression subcohort, Incidence subcohort, and Non-exposed control subcohort. OAI data and protocols are available for free public access^12^. This retrospective cohort study study includes 281 Caucasian individuals (129 male and 152 female) with an age range of 45–78 years (mean 58.48). Subjects included in the study were those whose telomere had already been quantified at baseline and whose results were already published in previous studies^10,11^. A subset was eligible based on availability of both baseline and 72-month radiographs and baseline PBL telomere length data. The OAI study was approved by the institutional review boards at each OAI clinical site and the coordinating center (University of California, San Francisco) and informed consent was obtained from the participants. This study was also approved by the local Galician Ethics Committee (*Comité Autonómico de Ética da Investigación de Galicia*) with registry code 2018/129.

### Evaluation of knee OA

X-ray images from both knees were considered in each individual, being evaluated according to the Kellgren-Lawrence (KL) score. KL grade was established for each knee, including patellofemoral and tibiofemoral joints. The KL grade assigned to the subject was the maximum obtained from the evaluation of the two knees, i. e. max-KL grade. *Prevalent radiographic knee O*A was defined when an individual had a maximum KL grade (max-KL) ≥ 2 considering both knees (n = 144), at recruitment. *Incident knee OA* was defined when max-KL grade increased from < 2 at recruitment to ≥ 2 after 72-month follow-up (n = 39). *Non-incident knee OA* was defined as max-KL grade maintained < 2 after the 72-month follow-up (n = 98).

### Evaluation of telomere sequences

DNA from PBL was extracted from the blood sample obtained at recruitment and after a 6-year follow-up. Coded DNA samples were processed by research personnel blinded to the status of the subjects. The average telomere amount in PBL was measured with a standard validated quantitative PCR (qPCR) based assay as described^16^. This method measures the average ratio of telomere repeat copy number to a single gene (36B4) copy number (T/S ratio) in each sample. The technique was performed using a LightCycler thermocycler (LightCycler 480, Roche Diagnostics, Werk Penzberg, Germany). DNA samples were amplified in parallel 20 μl PCR reactions that contained 10 ng of sample DNA, the DNA master SYBR Green I kit (LightCycler 480 Sybr Green I Master, Roche Diagnostics) and 500 nM of primers for the telomere (forward: 5′ CGGTTTGTTTGGGTTTGGGTTTGGGTTTGGGTTTGGGTT 3′; reverse 5′ GGCTTGCCTTACCCTTACCCTTACCCTTACCCTTACCCT 3′) and for the 36B4 (forward: 5′ CAGCAAGTGGGAAGGTGTAATCC 3′; reverse: 5′ CCCATTCTATCATCAACGGGTACAA 3′). To avoid possible inter-assay variability, the paired samples of each subject, at recruitment and after 6 years, were processed in the same plate of PCR, each in quadruplicate. The average efficiency was 1.8 for telomeric amplification and 1.9 for 36B4 amplification. The T/S ratio was calculated using these efficiency values: T/S ratio = efficiency^−CqTel^/efficiency^−Cq 36B4^. The relative percentage of telomere loss in each individual was defined as [(T/S ratio at recruitment − T/S ratio after 72 months) / T/S ratio at recruitment] × 100.

### Statistical analysis

Caucasian individuals (n = 281) were included in this study. This sample size allowed us to estimate a difference of 9% and 5% in the relative loss of telomere between the incident and non-incident population and, prevalent vs non-prevalent, respectively, with a safety of 95% and a statistical power of 90%.

Descriptive analysis was performed for all variables studied. Continuous variables were reported using means ± standard deviations (SD). For dichotomous/categorical variables, absolute numbers and percentages were computed.

Relative percentage of PBL telomere loss after 6 years was analyzed according to patients’ characteristics as gender, hypertension and baseline max-KL grade, using the Mann–Whitney and Kruskall-Wallis test. For age and BMI, the Spearman’s Rank Correlation Coefficient was performed.

A univariate and multivariable logistic regression analysis were performed to identify the variables independently related with prevalent and incident radiographic knee OA.

A multivariable logistic regression analysis was performed to determine the relative percentage of PBL association with incident OA, adjusting for gender, BMI and WOMAC as potential confounders. Odds Ratios and their 95% confidence intervals (CI) were expressed.

All of the tests were carried out bilaterally, considering values of p < 0,05 as significant. Data were analyzed using SPSS software version 20 (Chicago, Illinois, USA).

### Ethics approval and consent to participate

The OAI study was approved by the institutional review boards at each OAI clinical site and the coordinating center (University of California, San Francisco) and informed consent was obtained from the participants. This study was also approved by the local Galician Ethics Committee (*Comité Autonómico de Ética da Investigación de Galicia*) with registry code 2018/129. All research was performed in accordance with relevant guidelines/regulations and with the Declaration of Helsinki.

### Consent for publication

Non applicable.

## Data Availability

The datasets used and analyzed during the current study are available from the corresponding author on reasonable request. Data from the OAI is available at the Osteoarthritis Initiative Data Center (https://oai.epi-ucsf.org/datarelease/).

## Abbreviations

BMI: Body mass index
KL: Kellgren–Lawrence
max-KL: Maximum KL grade
OA: Osteoarthritis
OAI: Osteoarthritis initiative
PBL: Peripheral blood leukocytes
qPCR: Quantitative PCR
T/S ratio: Average ratio of telomere repeat copy number to a single gene (36B4) copy number
WOMAC: Western Ontario and McMaster Universities Osteoarthritis Index
